# To Push or To Pull? In a Post-COVID World, Supporting and Incentivizing Antimicrobial Drug Development Must Become a Governmental Priority

**DOI:** 10.1021/acsinfecdis.0c00681

**Published:** 2021-02-19

**Authors:** J. Cama, R. Leszczynski, P. K. Tang, A. Khalid, V. Lok, C. G. Dowson, A. Ebata

**Affiliations:** † Living Systems Institute, University of Exeter, Stocker Road, Exeter EX4 4QD, U.K.; ‡ College of Engineering, Mathematics and Physical Sciences, University of Exeter, Exeter EX4 4QF, U.K.; § Polygeia, Global Health Student Think Tank, London, U.K.https://www.polygeia.com/; ∥ Faculty of Life Sciences and Medicine, King’s College London, Great Maze Pond, London SE1 1UK, U.K.; ⊥ School of Clinical Medicine, University of Cambridge, Cambridge CB2 0SP, U.K.; # School of Biological and Chemical Sciences, Queen Mary University of London, Mile End Road, London E1 4NS, U.K.; ∇ School of Life Sciences, Gibbet Hill Campus, University of Warwick, Coventry CV4 7AL, U.K.; ◆ Antibiotic Research U.K., Genesis 5, York Science Park, Heslington, York YO10 5DQ, U.K.; ○ Institute of Development Studies, Library Road, Brighton BN1 9RE, U.K.

**Keywords:** antimicrobial resistance, global health policy, market failure, push and pull incentives, access, One Health

## Abstract

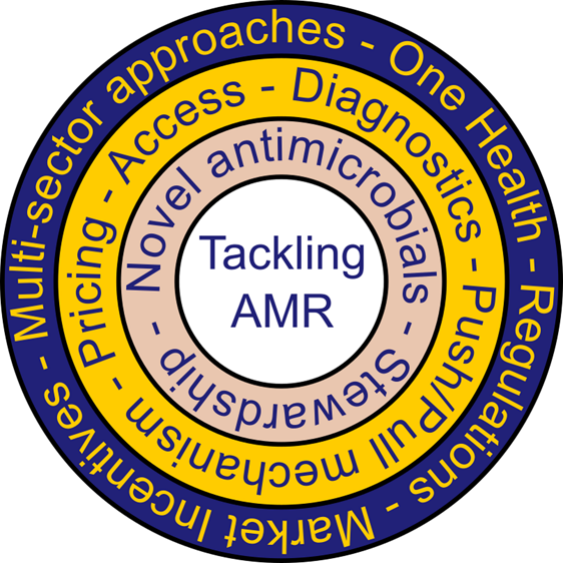

The COVID-19 pandemic has refocused attention worldwide on the dangers of infectious diseases, in terms of both global health and the effects on the world economy. Even in high income countries, health systems have been found wanting in dealing with the new infectious agent. However, the even greater long-term danger of antimicrobial resistance in pathogenic bacteria and fungi is still under-appreciated, especially among the general public. Although antimicrobial drug development faces significant scientific challenges, the gravest challenge at the moment appears to be economic, where the lack of a viable market has led to a collapse in drug development pipelines. There is therefore a critical need for governments across the world to further incentivize the development of antimicrobials. Most incentive strategies over the past decade have focused on so-called “push” incentives that bridge the costs of antimicrobial research and development, but these have been insufficient for reviving the pipeline. In this Perspective, we analyze the current incentive strategies in place for antimicrobial drug development, and focus on “pull” incentives, which instead aim to improve revenue generation and thereby resolve the antimicrobial market failure challenge. We further analyze these incentives in a broader “One Health” context and stress the importance of developing and enforcing strict protocols to ensure appropriate manufacturing practices and responsible use. Our analysis reiterates the importance of international cooperation, coordination across antimicrobial research, and sustained funding in tackling this significant global challenge. A failure to invest wisely and continuously to incentivize antimicrobial pipelines will have catastrophic consequences for global health and wellbeing in the years to come.

## Introduction

As we survey the damage caused by COVID-19, it is increasingly clear that societies and governments across the globe underestimated the ever present threats posed by infectious diseases.^1^ The widespread loss of lives and livelihoods that continues through the COVID-19 pandemic highlights the susceptibility of our deeply interconnected global networks to novel infectious agents and reminds us that infectious diseases respect no border, causing millions of deaths across both low-income^2^ and high-income countries (HICs) every year.

However, while the world focuses on a viral pandemic for the present, we must not forget the potentially even more disruptive burden of antimicrobial resistant (AMR) bacterial and fungal pathogens, which threaten the very edifice of modern medicine. Without changes in policy, it is estimated that antimicrobial resistant infections may result in 10 million *annual* deaths by 2050.^3^ Further, the World Bank estimates that the global economy may lose up to 3.8% of its annual gross domestic product (GDP) by 2050, with an *annual* shortfall of up to $3.4 trillion by 2030.^4^ Worryingly, the World Bank report itself suggests that this may be an underestimate, since the impact of AMR pathogens was modeled on the basis of shocks to labor supply and livestock productivity, which may not fully account for all the economic effects of AMR.^4^ Economic losses on such a scale will inevitably threaten public health and livelihoods across the globe.^5,6^

Modern medical systems have been developed assuming a continuous supply of functional antimicrobials to combat infectious disease and deliver effective health care. For example, without effective antibiotics, a wide range of common surgical procedures may be deemed too dangerous to perform due to the risk of potentially untreatable surgical site infections (SSIs),^7−10^ thereby massively decreasing quality of life for patients. Childbirth and caesarean sections will become fraught with danger for both mother and child;^11,12^ indeed, in 2016, a World Health Organization (WHO) commentary reported that approximately 200 000 newborns die annually due to infections that do not respond to existing drugs, highlighting the increasingly widespread burden of drug resistance.^13^ The scourge of tuberculosis has returned in multidrug resistant form, presenting an enormous global health challenge.^14^ Multidrug resistance has been detected in a wide range of common pathogenic bacterial species including *Klebsiella pneumonia*,^15^*Salmonella enterica* serotype typhi,^16^*Enterococci* spp.,^17^ and many others. The plague, cause of the worst pandemics known to history, has seen a resurgence in cases over the past couple of decades.^18,19^ In 1997, a multidrug resistant clinical isolate of *Yersinia pestis* (the causative agent of plague) was reported,^20^ and surveillance of antibiotic resistance in *Y. pestis* worldwide is now a necessity.^21^

Bacterial infections even exacerbate the health impacts of respiratory viral diseases such as influenza. Indeed, the majority of deaths in the 1918–1919 H1N1 influenza pandemic are believed to have resulted directly from secondary pneumonia caused by bacterial species commonly found in the upper respiratory tract; similar trends were observed in the viral pandemics of 1957 and 1968.^22^ The 2009 swine influenza pandemic saw secondary bacterial pneumonia identified in 29–55% of mortalities.^23^ Without effective antibiotics, deaths from secondary bacterial infections will inevitably increase with the burden of viral diseases. In the case of COVID-19, an early study reported that although only 6.9% of COVID-19 patients were showing bacterial co-infections, antibiotics (in most cases broad spectrum agents) were being given in over 70% of cases.^24^ Such overuse may exacerbate the problem of AMR, particularly in hospital settings.^25^

In this Perspective, we focus on the risks associated with growing bacterial resistance to antibiotics and the economic challenges underlying the failure of the development pipeline to tackle drug-resistant infections (Box 1). We examine different incentive schemes to reinvigorate antibiotic research and development (R&D) in the pharmaceutical and biotechnology industries. We compare and contrast various “push” incentives, which involve directly lowering the cost of R&D, with “pull” incentives that instead aim to improve revenue. We suggest that a range of pull incentives that address the antibiotic market failure crisis are needed to complement the push incentives currently supporting drug development. A *combination* of appropriate push and pull incentives, financed and supported at least initially by governments and tailored to the specific needs of individual developers, is required to circumvent this crisis. We discuss how any publicly funded schemes must incorporate broader “One Health” considerations regarding appropriate antimicrobial use and manufacturing practices to be truly effective in the long term. We also argue for simultaneous incentives for rapid diagnostic testing, and importantly its integration into clinical practice, to support the next generation of antimicrobial therapies and effective clinical decision-making. Finally, we support recent calls for the establishment of a supranational treaty to coordinate AMR efforts at a global level and suggest mechanisms to devolve specific responsibilities across HICs and LMICs (low- and middle-income countries) within such a treaty (Box 2).

Box 1 **Major Policy Challenges**

- Without changes in policy, antimicrobial resistant infections are predicted to cause 10 million annual deaths by 2050.^3^
- However, it is not only resistant infections that cause significant mortality. The lack of access to antimicrobials itself causes around 5.7 million deaths annually from infections that are currently treatable.^92^
- Market failure hinders the commercial development of new antimicrobials, despite recently increased “push” funding to cover R&D costs.
- In the absence of other incentives, new antibiotics must be priced significantly higher than older generic antibiotics to make their development commercially viable.
- Incentives are required to refresh the antibiotic pipeline, particularly to address clinically unmet needs. Any incentives must also be contingent on meeting access requirements, particularly in LMIC settings.
- Incentives are also required to support the clinical translation of rapid diagnostics to facilitate appropriate stewardship of antibiotic prescribing in clinical settings.
- Poor antibiotic manufacturing practices and agricultural misuse also contribute to the spread of resistance, requiring AMR specific policy making across a range of policy areas.
- The multifaceted problems of AMR require global cooperation across multiple policy areas, in a variety of different socioeconomic settings.

Box 2 **Proposed Policy Solutions**

- A range of “pull” incentives are required in addition to the current “push” incentives to solve the antimicrobial market failure problem and stimulate antimicrobial R&D pipelines.
- Additional push incentives are required to support early translational work, including structurally informed medicinal chemistry to develop clear antibacterial activity.
- Governments and payers must be allowed flexibility in the formulation of incentive packages; individual companies will likely require bespoke solutions, depending on their size, the number of drugs in their pipeline, and the number of drugs brought to market.
- Any commercial incentives must be tied to stewardship, the use of manufacturing standards to limit environmental contamination, and equitable access across both HICs and LMICs, depending on the clinical need.
- The incentive package should also be dependent on the quality of the new drug, with larger incentives for the development of drugs where resistance will take longer to develop. However, the speed of the development of resistance to a new drug is unpredictable, which suggests that some of the rewards for drug development along these lines may need to be held back until after the drug is on the market. The rewards would also need to be awarded in a phased manner. The recently proposed Antibiotic Susceptibility Bonus, which details conditional payments post-market-entry as part of MER incentives, offers a potential mechanism to address this problem.^106^
- Establish a global supranational treaty modeled on the Paris Climate Agreement to coordinate policy interventions and incentives across the globe, while ensuring equitable access to drugs depending on clinical need.
- Establish frameworks to facilitate open collaboration in basic research and clinical trials for antimicrobial development.
- Increase support to early career academics and doctoral training in the antimicrobial drug discovery field, particularly those developing innovative, novel approaches, to ensure that the antimicrobial development ecosystem is sustainable and to prevent the sudden collapse of the skills and talent pool in the field.

## Economic Disincentives for Antimicrobial Discovery

Despite the importance of antibiotics in the medical system, and the additional threat of the re-emergence of untreatable pandemic bacterial diseases, investment in developing new antibiotics remains neglected.^26^ While serious scientific challenges exist in antibiotic R&D,^27,28^ one of the gravest challenges lies in the economics of antibiotic development.^3,29−31^ The commercial success of a drug has, in the past, typically been dependent on a combination of its sales and price. The antibiotics market suffers from a unique set of problems in these two respects. First, higher sales volumes are more likely to drive the rapid emergence of resistance.^32^ Doctors are therefore encouraged *not* to prescribe new antibiotics unless absolutely necessary and to generally reduce antibiotic prescribing to slow the spread of resistance. This has led to a marked decrease in antibiotics sales over the past few years in countries such as the UK, which has a strong focus on antibiotic stewardship.^33^ Second, the prices of antibiotics in the market are also influenced by the abundance of low-price generics;^34^ for example, a 2015 study reported that the widely used antibiotic vancomycin was available in the UK for under £35 a day.^35^ However, the prices of new antibiotics are then often benchmarked against these low-cost drugs. For example, in the USA, by far the world’s largest pharmaceutical market, the diagnosis-related group (DRG) reimbursement system assumed that generic antimicrobials will be used to treat infections. Hospitals that required branded antimicrobials to treat resistant infections would lose thousands of dollars on each patient requiring such treatment, thus disincentivizing the addition of new antibiotics to their formularies and further exacerbating the market failure crisis.^36^ Important reforms in the reimbursement mechanisms targeting these artificial price caps on novel antibiotics were introduced by the Centers for Medicare and Medicaid Services (CMS) in 2019 to encourage innovation in the field.^37^ However, it remains to be seen whether this is enough or whether further reforms are required. Similar reimbursement related economics plague antibiotic pricing in Europe as well, but in a promising development, Germany has recently announced reforms to facilitate higher reimbursements for “reserve” antibiotics that are meant to be used only when treating multidrug resistant infections.^38^

These price-related difficulties for antimicrobials are in stark contrast to the pricing of new treatments for other disease indications like Hepatitis C, which cost closer to £30,000 per patient in the UK,^35^ or the latest generation CAR-T-cell (chimeric antigen receptor T-cell) cancer therapies that can reportedly cost hundreds of thousands of pounds. The high prices of these drugs are justified by the developers, and generally accepted (after some negotiation) by payers, based on their vast superiority over older treatments, despite access related concerns.^39^ However, in the case of antibiotics, a new drug is only vastly superior when used to treat an infection that is resistant to *all* available low-price generics. At present, pan-drug resistant infections are still relatively rare (although increasing in number^40^), making it difficult to justify pricing new antibiotics in a manner similar to new Hepatitis C or CAR-T-cell therapies. To sustain innovation in antibiotic development, financial modeling suggested that the price of a new antibiotic, assuming a treatment time of 2 weeks, would need to be approximately $1,000 per day in the absence of other incentives to make the returns viable for the developer;^41^ we are indeed seeing prices in this range for new products such as ceftazidime–avibactam for certain indications.^42^ However, even these prices may not be enough to reinvigorate the pipeline. For example, Avycaz (the brand name of ceftazidime–avibactam), approved in 2015 by the FDA for complicated intra-abdominal and urinary tract infections, had sales of $43 million in the first 9 months of 2017,^43^ which is likely still too low to interest private investors, given the risks and costs associated with development. Counterfeit, substandard, and falsified antibiotics, encountered since the introduction of penicillin, further exacerbate these problems.^44,45^

The upshot of this is that although the societal benefit of new antibiotics is very high (the monetary benefits to society of a new antibacterial are estimated to range between $486 million and $12 billion depending on the indication^46^), the modeled “private” value (i.e., the value for the drug developer) ranges from *negative* (−$4.5 million) to positive $37.4 million.^35^ In contrast, the so-called net present value (NPV) of a new arthritis drug is estimated to be positive $1 billion at discovery.^29^ This commercial reality has led to a complete failure of the antibiotic market, and most big pharmaceutical companies have abandoned the field and diverted resources into more profitable products. This has left the bulk of the antibiotic R&D mantle in the hands of small biotechnology companies.^47−49^ However, as the recent bankruptcies of Achaogen and Melinta demonstrate, even when companies successfully manage to develop antibiotics and bring them to market, the cost of discovery and development, combined with the lack of a profitable market, leads to their collapse. This in turn dampens investor confidence, discouraging further investment.

This is already beginning to have severe consequences. The O’Neill report commissioned by the UK government estimated in 2016 that 700 000 people a year die due to antimicrobial resistant infections. As mentioned previously, projections suggest that, without intervention, the annual death toll would reach 10 million by 2050.^3^ The burden of drug resistant infections is disproportionately greater for LMICs at present,^50^ but as we have seen with the COVID-19 pandemic, emerging infectious diseases are a *global* threat. It is a question of when and not if these pathogens spread across the globe, as has been seen previously, for example, with the rapid spread of pathogens hosting the NDM-1 multidrug resistance gene across the world from its origin in India.^51^ New antimicrobials will inevitably be required across the globe, not just in LMICs.

It is therefore critical, even as the world counts the costs of the COVID-19 pandemic, to urgently resolve the market failure problem of these crucial drugs.

## Pull Incentives Are Needed to Complement Existing Push Funding Mechanisms to Accelerate the Development of Antimicrobials

Over the past decade, several incentives have been proposed to encourage the pharmaceutical industry to re-engage in antimicrobial R&D. These can be broadly categorized into two groups: push incentives and pull incentives (Table 1).

**Table 1:** Examples of Push and Pull Incentives with Their Advantages, Disadvantages and Relevance for SMEs and Large Pharmaceutical Companies

incentives	advantages	disadvantages	SMEs vs large pharmaceutical companies
**Push Incentives**^b^			
direct funding and support	• directly lowers initial R&D costs	• less suitable for late-stage R&D	• allows SMEs to overcome initial cost of the R&D pipeline
	• expert technical support, planning, and assistance	• high risk of R&D failure; risk borne by funder	• helpful for SMEs with less experience
			
tax incentives	• lowers the whole R&D pipeline cost	• less transparency than direct funding	• SMEs less likely to benefit on the whole compared to large pharmaceutical companies due to lower revenue gains
	• such mandates are familiar to governments	• financial risk of R&D failure is borne by the government	• however, tailored tax incentives may be more beneficial for SMEs; for example, tax incentives based on payrolls might be more beneficial than those linked to income tax, particularly for start-ups, which typically do not have income tax liabilities^52^
			
partnerships	• risk of R&D failure spread between all stakeholders	• financial risk of R&D failure is borne by the stakeholder with largest share or investment	• allows SMEs to overcome initial cost of the R&D pipeline
	• nonprofit partnerships (e.g., NGOs) not interested in maximizing sales profit	• decisions require approval and discussion from all stakeholders; time-consuming	• helpful for SMEs with less experience
			• large pharmaceutical companies likely to engage for high-risk R&D antimicrobial projects
**Lego-regulatory Pull Incentives**^c^			
market exclusivity extensions	• R&D costs recouped beyond the initial patent life-span	• may dampen competition/innovation	• large pharmaceutical companies likely to benefit more than SMEs, due to higher chances of successfully marketing novel antimicrobials; however, SMEs may also benefit equally if the extensions are made transferable, such that they may be traded or sold
	• reduces the need to inappropriately drive sales and therefore decreases antimicrobial misuse	• extended patents can delay cheaper generics entering the market	
			
accelerated approvals	• speeds up R&D pipeline	• risk of safety and efficacy, as novel antimicrobial is expedited	• SMEs less likely to benefit than large pharmaceutical companies due to being less likely to reach the late-stage R&D pipeline (phase 2/3 clinical trials) and also due to fewer drugs in the pipeline; may be effective for SMEs when combined with appropriate push funding
	• reduces cost of initial and prolonged R&D pipeline		
			
tradeable exclusivity vouchers	• promotes successful novel antimicrobial R&D despite low sales	• exchanged vouchers for exclusivity of more expensive drugs treating noninfectious diseases might become an ethical issue	• large pharmaceutical companies will benefit since they are likely to already have an established highly profitable drug treating a noninfectious disease to which the voucher may be applied
			• SMEs may not have other drugs in-house to which these vouchers would be applicable; however, they will benefit by auctioning the vouchers to other companies; the level of benefits gained will be subject to the outcome of such sales and negotiations with other companies
**Reward-Based Pull Incentives**^d^			
one-off monetary prizes	• promotes successful antimicrobial development through late-stage R&D	• risk borne by SMEs or pharmaceutical industry	• SMEs less likely to benefit than large pharmaceutical companies due to high initial cost of R&D, if this incentive is applied in isolation; however, when combined with an appropriate push incentive, chance for synergizing the advantages of both incentives to benefit SMEs
	• easy to implement via NGOs or governments	• difficulty in setting the optimal value of the reward	
			
milestone monetary prizes	• promotes successful antimicrobial development through late-stage R&D	• difficulty in setting the optimal value of the reward	• SMEs supported throughout the R&D pipeline if successful
	• easy to implement via NGOs or governments	• funding risk if R&D fails	• large pharmaceutical companies likely to engage for high-risk R&D antimicrobial projects
			
market entry rewards (can be either fully or partially delinked as detailed below)	• promotes successful antimicrobial development through late-stage R&D	• risk borne by SMEs/pharmaceutical industry	• SMEs less likely to benefit than large pharmaceutical companies due to high initial cost of R&D, if the incentive is applied in isolation; however, SMEs will benefit when MERs are combined with appropriate push incentives to cover R&D costs
		• difficulty in setting the optimal value of the reward	
			
fully delinked market-entry rewards	• all revenue would be from market entry reward and antimicrobials could be sold at a minimal cost	• cost of sustaining is greater than the partially delinked model since no revenue is generated from sales	
	• no incentive to overmarket, thus helping with stewardship		
			
partially delinked market entry rewards	• revenue generated would be split between market entry rewards and sales, which may be more sustainable than a fully delinked award since the costs would be lower	• could encourage overselling as still partially linked to units sold	
	• may be used with existing reimbursement mechanisms		
	• price may still be controlled to facilitate access		

a Information in this table has been adapted from Renwick et al. (2016),^53^ Årdal et al. (2017),^54^ Morel and Mossialos (2010),^30^ Mossialos et al. (2010),^31^ and the DRIVE-AB report.^34^ NGOs = nongovernmental organizations; R&D = research and development; SMEs = small and medium-sized enterprises; MERs = market entry rewards.

b Push incentives are associated with directly lowering the cost of research and development.

c Lego-regulatory pull incentives are associated with policies aimed at indirectly improving revenue generation.

d Reward-based pull incentives are associated with providing revenue generating benefits after successful research and development.

**Figure 1:**
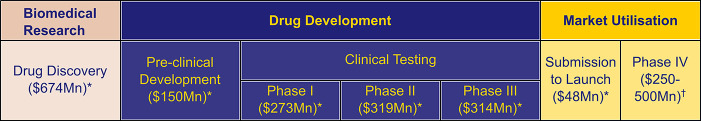
Summary of the different R&D stages and pharmaceutical industry costs involved in the development of a new therapeutic. Estimates of costs are taken from Paul et al. (2010)^55^ (marked *, modeled capitalized costs per launch of a new molecular entity) and Rex 2020^56^ (marked †, specifically for antibiotics for the first five years postapproval). Recent estimates put the capitalized cost of bringing a new antibiotic to market at around $1.3 billion.^56,57^ However, a figure that is often overlooked is the cost *postapproval*, which for antibiotics is estimated to be $250–500 million over the first five years that the drug is on the market.^56^

Push incentives are defined as strategies associated with directly lowering the costs of *developing* a new antimicrobial drug candidate. This outcome can be achieved by distributing the expenditure among multiple stakeholders, which reduces the economic risk associated with the failure of a potential drug candidate. As such, push incentives can be seen as early funding that can assist pharmaceutical companies in progressing through the different R&D stages (Figure 1) or as an incentive that partly offsets the costs of a potential project failing; this includes scientific research grants or direct funding.

Pull incentives, on the other hand, reward those who successfully develop a novel antimicrobial by increasing or ensuring future revenues. More specifically, pull incentives can be further subcategorized into either outcome-based or lego-regulatory. Outcome-based pull incentives are associated with advanced milestone reward payments, which can be given at each successful R&D stage, as well as at the market utilization stage (Figure 1). Lego-regulatory pull incentives are associated with policies that indirectly enable greater returns for the developer in the future, such as market exclusivity extensions.

Due to the inherent challenges in drug development and high failure rates, push incentives cover both unsuccessful projects and successful therapeutics. However, although *crucial* for early stage development, these incentives do not reward successful drug development at the late R&D stage of market entry, as these incentives do not guarantee profits. For example, Achaogen received funding to enable the development of its new aminoglycoside plazomicin up to commercialization, but the company still collapsed postapproval due to insufficient sales that could not offset the costs incurred. This highlights the fact that push incentives alone are unlikely to sustain antimicrobial pipelines; the added use of *pull incentives* is required to prevent further such bankruptcies. The UK has taken a lead on this approach and will be trialing a subscription system with two contracts to pay pharmaceutical companies up front depending on the usefulness of the therapeutic, rather than relying on sale profits to drive investment.^58−60^

Market entry rewards (MERs) are examples of outcome-based pull incentives, with monetary rewards provided when the antimicrobial enters the market. These may be fully delinked, where the reward is not proportional to the units sold, or partially delinked, where part of the revenues still come from sales of the product. In terms of cost, the partially delinked model is considered by some to be more sustainable,^54^ as in this model a portion of the money is generated through sales. Besides the lower costs, the partially delinked models may also be used in conjunction with existing reimbursement mechanisms.^54^ However, on the flip side, as there is still some dependence on sales, this again creates an incentive to maximize sale volumes, thus impacting stewardship. Such partially delinked rewards will need careful fine-tuning in terms of balancing the reward payments and expected market returns to ensure that the new drugs funded by these mechanisms are not overused, while ensuring that the risk in investment borne by private entities is also appropriately balanced against potential rewards.

The delinkage model has the added benefit of having a lower likelihood of secondary disruptive effects compared with other pull incentives.^61^ This is because, unlike some other incentive models, such as market exclusivity extensions, they do not impact patient drug access either through increased pricing or delayed generics. However, this comes at the cost of relying on sustained funding. For both the fully delinked and partially delinked MER models, it is necessary to consider how these rewards would be funded. The O’Neil report provides an example of using a “pay or play” tax that could provide funding, with companies that choose not to invest in antimicrobial research paying an additional tax. However, the implications on the cost of medicines because of this tax may then have knock-on effects for access to other drugs produced by these companies.

On the other hand, tradeable exclusivity vouchers are an example of a lego-regulatory pull incentive, which enable companies producing antimicrobials to extend the exclusivity period of another more profitable drug.^54^ Indeed, various types of vouchers have been proposed including priority review vouchers, where another more profitable drug may be awarded a priority review status and fast-tracked in the review process, enabling quicker market entry. However, there are several limitations to such a scheme^62^ that might impede patient access to treatments for other conditions. For example, if there is a delay in access to generics for other conditions such as cancer, due to an extension of the exclusivity period for a cancer drug, this might limit patients being able to access these treatments. Carefully thought out solutions, such as ensuring that the value of a voucher is tightly coupled to the value of the antimicrobial developed, will be crucial to the success of such a pull mechanism.^62^

Strikingly, over 95% of antimicrobial drugs in development today are from *small* companies, and approximately 75% of these companies are “pre-revenue” with no products on the market.^63^ To build a sustainable pipeline of antimicrobials in the future, we need to both support the SMEs currently driving antimicrobial R&D and also attract “Big Pharma” back into this space. Big Pharma has the advantage of access to expertise across a wide variety of fields, such as medicinal chemistry, pharmacology, and (in the case of antimicrobial R&D) microbiology that are critical for the success of drug discovery programs, especially when the classes of drugs being discovered are novel. Further, the discovery of novel antimicrobial classes will require fundamental exploratory research, potentially easier in a Big Pharma setting than in an SME where the pressures of external financiers and tight timelines may disincentivize much exploratory work. They also have significant lego-regulatory and sales infrastructure in place enabling the smooth transition from discovery and development to revenue generation. By comparison Achaogen had to restructure 28% of its workforce away from primary R&D to support the sale of plazomicin. That said, Big Pharma also suffers from timeline/milestone disincentives that may derail early stage exploratory projects. Closer interactions between pharmaceutical companies and academia in the field may be a potential solution to this problem, although more flexible and timely mechanisms of funding would be required to enable this, potentially through a national consortia with expert antimicrobial discovery oversight, enabling the rapid progression of promising projects. Since SMEs often involve spin-outs from academic research groups, a close working collaboration spanning academia, SMEs, and Big Pharma may facilitate the discovery of novel classes of antimicrobials, which are desperately needed. Government funded nonprofit organizations such as the Medicines Discovery Catapult in the UK ought to be well placed, with appropriate targets and support, to facilitate these interactions, given their unique role in the drug discovery ecosystem.

Given the different scales and financial capabilities of the partners needed to reboot antimicrobial discovery, incentive strategies will need to be flexible, to address the specific needs of individual companies (we have compared the advantages and disadvantages of various incentives from the perspective of SMEs vs large pharmaceutical companies in Table 1). This could include using a hybridization of multiple pull incentives or a combination of push and pull incentives. An example of a proposed hybrid strategy is the Options Market for Antibiotics (OMA), which allows investment in the development of a drug in return for receiving a specified number of units of the drug at a reduced price, if and when the antibiotic successfully reaches the market.^64^ In addition to receiving the drug at a reduced price on market entry, the option purchaser also drives the development of the drug that they desire; this may be of interest to funders or investors who wish to target specific pathogens that are a burden in LMICs, for example, where the market conditions for a new drug are especially challenging.^64^ Another example proposed as part of the Antibiotic Conservation Effectiveness strategy hybridizes outcomes-based and lego-regulatory pull incentives by using a combination of conservation-based market exclusivity, antitrust waivers, and value-based reimbursement.^65^ It is worth mentioning that such hybrid strategies may be of interest for incentivizing the development of drugs for neglected tropical diseases, which also suffer from a lack of investment from drug developers due to market considerations.^66^

## Incentivizing “Non-antibiotic” Therapeutics

Given the difficulties facing the development of traditional antibiotics over the past few decades, attention is now also turning to alternative “non-antibiotic” therapeutics as a means of addressing the AMR challenge.^67^ A recent WHO review reported that over one-third of antibacterials in preclinical development were nontraditional products. It is therefore worth considering how incentive strategies might need to be adapted to support these novel therapeutics.^68^ Funding to specifically incentivize nontraditional approaches has been provided by CARB-X in the recent past,^69^ but additional incentives will be needed to sustain development through to market, via the pull incentives in the previous section.

Nontraditional antimicrobial therapies have previously been categorized into four groups: standalone (e.g., phages, lysins, vaccines), transformations (e.g., Gram-negative activity achieved by combining polymyxin B analogues with approved Gram-positive antibiotics), augmentation (e.g., virulence factor inhibitor + approved antibiotic), and restoration (β-lactam + β-lactamase inhibitor combination).^67^ Given these often work alongside traditional antibiotics, regulatory approval may be challenging when the benefit is incremental and their benefit might only increase once resistance to the traditional antibiotic occurs.^70^ Clear regulatory guidance will therefore need to be given to companies developing these products, to ensure that this is not a barrier to their development and that there is clarity on the requirements in clinical trials to meet regulatory approval. Besides combinations of novel nontraditional therapeutics with older antibiotics, developers may also wish to consider using novel antibiotics (and especially novel classes, if and when developed) in combinations to prevent the rapid emergence of resistance that occurs, particularly when using single-target drugs. Such combination regimens are used, for example, in treating tuberculosis and HIV patients.^71^ However, developing therapeutic drug combinations is a pharmacological challenge. This will require a high-level of coordination and cooperation between developers of novel antibiotic classes, and a cost-effective regulatory pathway that facilitates the development of combination therapies for multiple novel classes of drugs.

As a corollary, our most effective antibiotics multitarget, inhibiting two or more enzymes (e.g., β-lactams and fluoroquinolones) or binding to components of 30S and 50S subunits that are multiply encoded (e.g., macrolides, aminoglycosides, tetracyclines) or inhibiting substrates encoded by biochemical pathways (e.g., glycopeptides). These antibiotics are generally resilient to resistance emerging by point mutation. This general principle must not be lost in the rush for novel approaches.

Companies developing nontraditional antimicrobials are also likely to be academic spin-outs and biotechnology companies who do not usually have industry-level expertise in drug development. Therefore, investment in the sharing and development of these skills from larger companies will be essential in driving the development of these alternative therapies.^72^

Despite an emerging and encouraging pipeline, there are relatively few “non-antibiotic” therapies currently on the market, which makes it challenging to identify the most appropriate incentive mechanisms for these products. It will be important to monitor the development and success of these therapeutics and consider their position in the overarching goal of combatting AMR, so as not to miss out on crucial opportunities. This is also an area that requires more fundamental research to overcome scientific barriers and may benefit from grant based push incentives for translational projects still anchored in academia. On a positive note, it is possible that private finance may look more kindly on investments in innovative antimicrobial approaches as compared to traditional small molecule antibiotics, especially when put in the context of the drive toward personalized medicine.^73^ Public–private partnerships may be a particularly interesting modality for developing these nontraditional products, which should be modeled further as the current preclinical pipeline matures. However, the issues around pricing and revenue remain similar for nontraditional and traditional therapies: if the patient group to be treated (i.e., sale volume) is small, challenges around revenue generation will remain for nontraditional products as well.

## The Clinical Translation of Rapid Diagnostics Must Also Be Incentivized to Support Antimicrobial Development

When debating the use of different incentives for stimulating antimicrobial development, one must also take into account wider problems in antimicrobial R&D and use. For instance, the recent WHO review of the preclinical antibiotic pipeline suggests that around 40% of these candidate therapies under development are pathogen-specific.^74^ Although this trend is promising and must be encouraged to lessen the impact of cross-resistance caused by the use of broad spectrum drugs,^75^ the clinical use of such drugs will be hindered by the lack of accompanying diagnostics that can clearly and quickly identify the infectious agent, including in low-income settings.^76^ This actually increases the commercial risks involved in the development of pathogen-specific therapies; these early stage projects may therefore require additional incentives. However, the lack of clinically available diagnostics is not just a problem for future therapies–for example, it is estimated that over 30% of antibiotic prescriptions in outpatient settings in the United States may be unnecessary,^77^ further driving the spread of drug resistance. This is intimately connected to the lack of clinically available diagnostics that can *rapidly* identify the disease causing pathogen and its corresponding antibiotic susceptibility profile.^78^ Importantly, such diagnostics need to clearly identify the disease causing pathogen, which is often technically challenging: infections may occur in locations that are difficult to access and sample or may involve multiple pathogens that may confound the diagnosis. That said, much can be done to improve upon best practice and compliance in the use of blood culture systems to identify pathogens for minimum inhibitory concentration testing, both within developed and developing health care settings.^79,80^ Further, the most common conditions where antibiotics are inappropriately used involve upper respiratory tract infections^81^ that are relatively easy to sample, and clinically available rapid diagnostics may play an important role in reducing unnecessary prescribing in these indications.

A number of rapid diagnostic technologies already exist, but clinical uptake and commercialization remains the challenge.^78^ An encouraging development in this regard is the recent investment by the European Union’s Innovative Medicines Initiative in the establishment of VALUE-Dx, a multidisciplinary consortium involving both industrial and nonindustrial partners, to develop and foster the clinical translation of rapid diagnostics to guide antibiotic stewardship (https://value-dx.eu/index.php/what-is-value-dx/). As with the rest of this field, the investment must be made now: debates about cost-effectiveness of widespread diagnostic testing at present, especially in publicly funded health systems, must bear in mind the need for supporting this diagnostic development ecosystem for the *future*, when it may urgently be required. Studies on the intergenerational ethics of AMR have stressed the importance of managing the problem bearing in mind that this is a slowly emerging disaster, which extends beyond the term of office of individual governments and requires visionary policies to build resilience and preparation for a world where few effective antimicrobials are available.^82^ We note that in the event of a fast spreading, drug resistant bacterial or fungal pathogen epidemic, rapid testing will be a priority; this is best exemplified by the critical need for rapid tests for the SARS-CoV-2 virus in the current pandemic and the difficulties that most countries have faced due to the lack of resilience and capacity in testing infrastructure.

## Incentives Should Be Linked to “One Health” Considerations to Ensure the Sustainability of New Antimicrobials

Another important aspect is the broader consideration of “One Health”^83^ in tackling AMR. A major point of delinking the value of new antimicrobials from the volume of sales is to protect the new therapeutic from overuse, inappropriate use, and accompanying resistance. However, poor manufacturing protocols can lead to environmental contamination with the active pharmaceutical ingredient, which leads to environmental reservoirs of resistance. Although at first glance one might only consider this to be a problem with generics manufacturers as we detail below, the lack of transparency in reporting environmental contamination with antibiotics is a problem with manufacturers across the globe. As reported by the ReAct group in 2018, only GlaxoSmithKline, Johnson & Johnson, Pfizer, and Roche had applied limits to antibiotics in effluent at supplier sites, and no companies had committed to publishing environmental audit results or antibiotic discharge levels.^84^

Recognizing the importance of the issue, steps have been taken at a global level to develop appropriate guidelines for producers of antibiotics. The WHO proposed various policies to guide both manufacturers and procurers of antibiotics in 2019.^85^ The AMR Industry Alliance has published “predicted no-effect concentrations” that may be used to establish antibiotic waste discharge targets at manufacturing sites.^86^ The Alliance has also established a manufacturing framework to guide responsible antibiotic production practices.^86^ However, much remains to be done to ensure the *implementation* of these policies globally. For example, in India, a major producer of antibiotics, recent reports suggest that the concentration of ciprofloxacin in treated wastewater from a pharmaceutical factory in the city of Hyderabad was equivalent to that required to treat 44000 people.^87^ Taking cognizance of the issue, the Indian government has proposed a law to tackle pharmaceutical pollution linked to antibiotic production, with standards even more stringent than those suggested by the AMR Industry Alliance.^88^ This is welcome news and represents the first attempt by a state regulator anywhere in the world to introduce such standards.^88^ However, the country’s pharmaceutical industry is already attempting to weaken the provisions of the law,^89^ and it remains to be seen how effectively any new limits on antibiotic waste will be enforced.

Similarly, inappropriate use of antibiotics in livestock farming has led to widespread bacterial resistance against critical antibiotics. As economies in many LMICs develop, demand for animal protein has rapidly increased.^90^ As a result, livestock farming is intensifying, leading to an increase in antibiotic use in feed and for therapeutic purposes. In Vietnam, for instance, critical antibiotics for humans including colistin, neomycin, and gentamicin were regularly used in pigs and chickens, leading to *Escherichia coli* strains developing resistance toward these antibiotics.^91^ If antibiotic use in farmed animals is not effectively regulated and restricted, developing new antibiotics for therapeutic use is unlikely to bring long-lasting benefits to human and veterinary health care.

## Global Coordination Is Key to Tackling the Diverse Challenges of AMR

**Figure 2:**
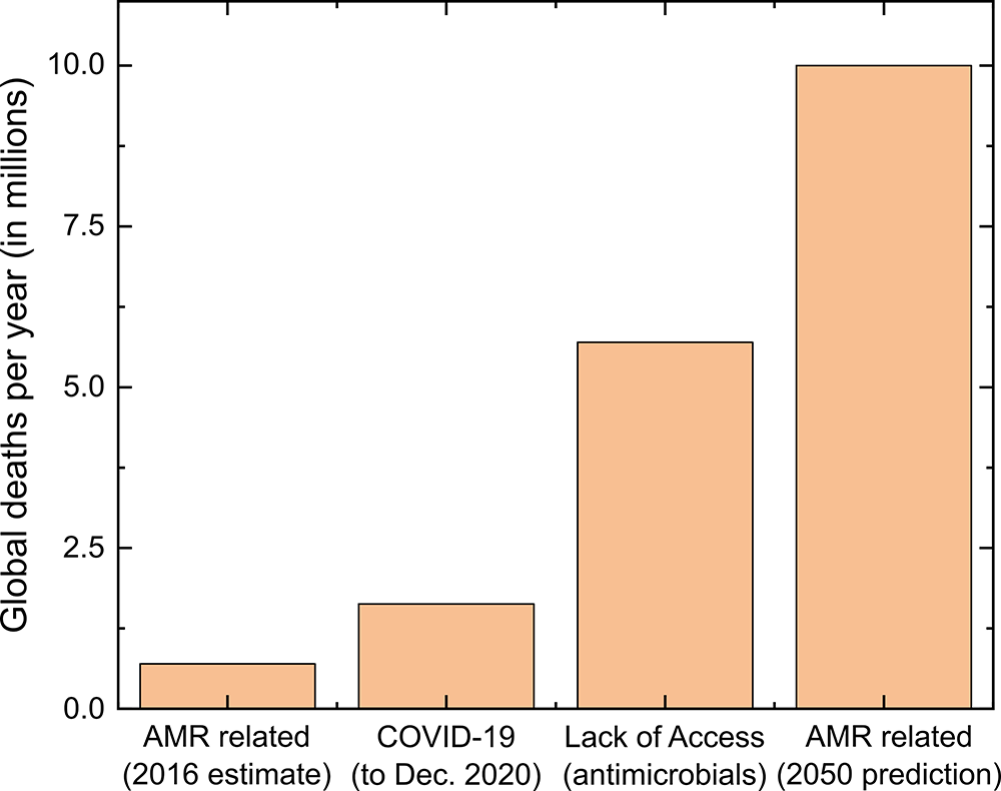
Estimates of global deaths due to AMR, including future predictions, and deaths due to treatable infections caused by lack of access to effective antimicrobials. Data based on the O’Neill (AMR related) and CDDEP reports.^3,92^ Current COVID-19 related deaths are provided for context, approximately a year into the crisis; data retrieved from Worldometer on 15th December 2020.

Our analysis shows that AMR is a multifaceted problem that affects populations across the globe. Further, AMR is an evolutionarily driven process, and not a problem that can simply be “solved” once and for all. Thinking beyond the problems of antimicrobial *resistance*, perhaps as troubling is the fact that 5.7 million people currently die annually of *treatable* infections, due to a lack of access (Figure 2) to effective antimicrobials.^92^ Therefore, governments and other public or charitable funders in particular will require strict eligibility criteria to select the beneficiaries of any proposed commercial incentives to ensure that public health, and specifically patient needs, are addressed at a global level.^62^ Robust health economic analysis and clinical considerations such as addressing unmet needs and focusing on drugs that are resilient to the development of resistance (Box 2), should be used to ensure that we incentivize the most beneficial antimicrobials; tying this tightly to regulatory conditions and access requirements will ensure that tax payers and patients receive value for their investments. It is important to note that these considerations may be viewed as further *disincentives* to antimicrobial R&D by the pharmaceutical industry, since they may pose additional liabilities on the developers. It is therefore crucial to address these issues at the earliest stages of negotiations between governments and charities (who will most likely pay for the incentives) and developers. These additional, and very important, costs must be factored in to the incentive packages to ensure that antimicrobial R&D is suitably incentivized while still providing the best value for the public investments proposed.

It is clear that these challenges would benefit from collective multinational action. At the CeBIL Annual Symposium in September 2019 in Cambridge (UK), on “legal innovations to support the development of anti-microbial drugs”, a discussion arose outlining the need for the AMR equivalent of the Paris Climate Accord. This idea is increasingly gaining traction, and the intervening months have seen detailed proposals put forth in a recent paper by Steven Hoffman and colleagues.^93^ The paper describes how certain unique features of the Paris Agreement could be reused in the development of a global AMR treaty, including the use of individualized national action plans on a country-by-country basis and the development of clear overarching goals that the treaty should target.^93^

**Figure 3:**
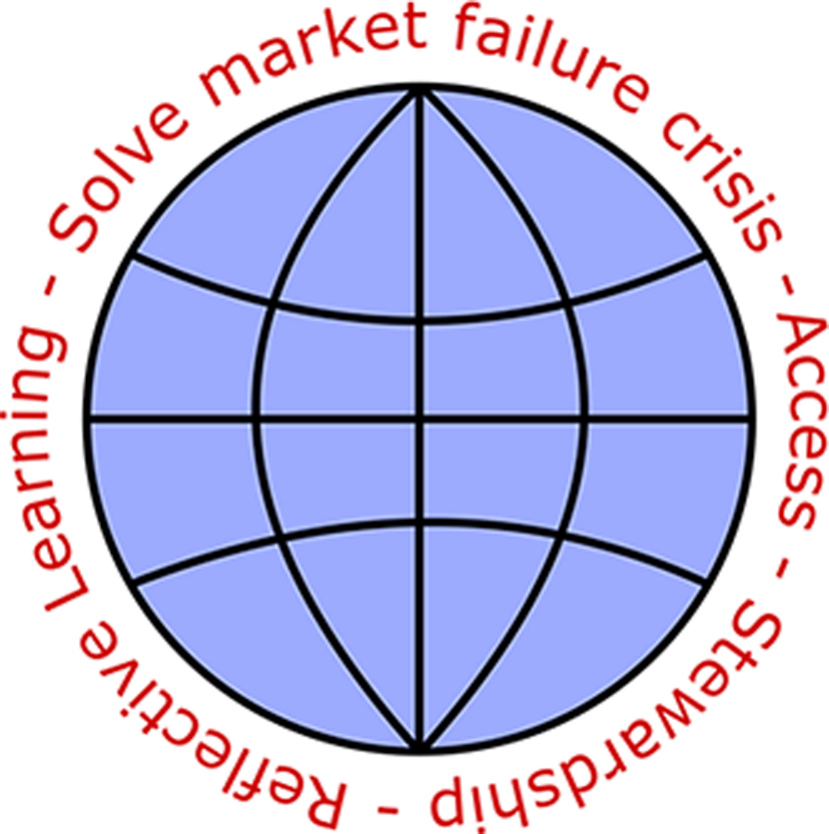
Global coordination is needed to solve the market failure crisis in antimicrobials and to balance access versus stewardship requirements across the globe. Continuous dialogue and reflective learning across nations will be critical for combatting the myriad policy challenges of AMR at a global level.

We believe such a treaty would facilitate the coordination of a global incentive plan, not only to develop, manufacture, and distribute new antimicrobials where they are most needed but importantly also to ensure the strictest adherence to suitable manufacturing waste discharge targets and the responsible use of the new drugs. This may also involve the establishment of a global repository for novel antimicrobials, with access provided to countries that abide by the treaty. Indeed, our analysis based on a literature review leads to three clear global goals that the treaty may address, which would devolve responsibility^93^ pragmatically across HICs and LMICs, as resources and capabilities enable (Figure 3):

(a) Commit to solving the antimicrobial market failure problem and incentivizing the development of one new antimicrobial every year that specifically addresses an unmet clinical need. HICs form the markets of greatest interest to pharmaceutical companies and are best placed to deliver this target, where possible in partnership with LMICs.
(b) Commit to leading the development of stringent, scientifically approved stewardship guidelines, particularly around waste discharge targets in antibiotic production and stewardship in clinical, veterinary, and agricultural use. As balancing access versus stewardship is a greater challenge in LMICs than HICs, context-specific laws and policies in this area may be better suited for development in LMICs, before being applied globally.
(c) Continuous dialogue and reflective learning between HICs and LMICs should be established to tailor policies that incentivize antimicrobial discovery and stewardship, to address not only growing resistance and the shrinking reserve of effective antimicrobials but also limited access to effective and affordable therapies particularly in low-income settings in LMICs.

In relation to point a above, given the magnitude of costs needed to incentivize antimicrobial development, it is imperative that governments in HICs bring together finance and health ministries to help lever the resources required. The proposed treaty may provide an umbrella under which these efforts could be pooled. One potential mechanism that might be suited to the task is the antibiotic Health Impact Fund.^94^ This would be funded by national governments (potentially also including major charitable organizations such as the Gates Foundation) and provide resources to drug developers who would in return register their product and receive regular reward payments proportional to (and conditional on) the clinical value of the product. The developer would agree to certain conditions involving the sale and distribution of the product, specifically addressing access related concerns across both HICs and LMICs. The fund would serve as a global coordination mechanism for developing new antibiotics and crucially would incentivize the appropriate timing of market entry for these drugs, thus addressing various resistance and stewardship related challenges that are inherent to the sale and use of new antibiotics.^94^

Further, with respect to point b, efforts to foster stewardship in LMICs are already in place. The Global Antibiotic Resistance Partnership (GARP), “ReAct”, and the “Alliance for the Prudent Use of Antibiotics” are examples of such efforts, focusing on understanding the spread of antibiotic resistance and effective policy responses to AMR. Successful stewardship will require going beyond AMR surveillance and addressing the constraints in health systems in LMICs.^95^ This includes expanding diagnostic capacity in clinical and community settings, continued education on AMR for both health care workers and the general public, increased regulatory capacity at the national and international levels to enforce regulations, and improving dialogue and collaboration between the public sector, private sector, and civil societies.^95^ We argue that working toward global antimicrobial stewardship requires responsible antibiotic use, as defined in Dyar et al.,^96^ needs to be linked to One Health, and needs to be extended to public policies that aim at incentivizing drug discovery.

These three recommendations will help HICs benefit from appropriate global stewardship and the corresponding reduction in drug resistant species, whereas LMICs would benefit from the new drugs bankrolled by HICs at affordable prices as long as strict stewardship criteria are enforced for their use. A dialogue between HICs and LMICs will ensure that drug discovery policies in HICs reflect the challenges of affordable access to effective antimicrobials in LMICs by, for instance, prioritizing therapeutics that can benefit a large proportion of populations that currently lack access.

Beyond an international treaty, which may take some years to organize, another means of facilitating global cooperation on AMR would be through the incorporation of AMR specific indicators in the Sustainable Development Goals (SDGs), as has recently been proposed by the ReAct group.^97^ Beyond tracking resistance and access related issues, discussing the need to reinvigorate the antimicrobial pipeline in a sustainable manner is, we argue, a worthy addition. Besides influencing and guiding high-level policy making, such supranational treaties and goals can be leveraged for attracting widespread public engagement with the problem and should include the further development of initiatives such as the World Antibiotic Awareness Week.^98^ A general public recognition of the value of antimicrobials, which may evolve in response to such supranational treaties, will be crucial for influencing political decisions regarding pharmaceutical incentives and antimicrobial stewardship in individual countries.

## Conclusion

In March 2020, while the global health community was focused on COVID-19, the discovery of yet another novel antibiotic resistance gene (garosamine-specific aminoglycoside resistance, *gar*) was reported.^99^ The gene provides high-level resistance against aminoglycosides; worryingly, it is suspected of having activity against plazomicin, the new antibiotic developed by Achaogen to circumvent the most common aminoglycoside resistance mechanisms.^99^ Although discovered in environmental samples from India, the gene was subsequently identified in environmental samples from Europe, Asia, Africa, and Australia and in a small number of clinical and food borne pathogen isolates in Europe, Asia, and North America, showing that the resistance gene had already spread globally across multiple pathogenic species, even before identification.^99^

This reiterates the fact that AMR is a global burden, which needs global solutions that can address the health, economic, scientific, political, and regulatory aspects of the problem. The current global ecosystem for incentivizing antimicrobial development has focused mostly on push incentives to fund drug development. We argue that this is insufficient in the face of the market failure problems: at a global level, a policy shift is required to reward antimicrobial drug development and production while still strictly regulating the usage of newly developed therapeutics. As we have outlined, a number of strategies already exist for this purpose, and governments need flexibility to develop bespoke incentive packages for different developers, which may be very different depending on the size of the company involved and its portfolio. This must also include incentives for the development of diagnostic tests to accompany new drugs that target specific pathogens; in particular, the use of these tests in clinical settings must be incentivized to maintain the test development ecosystem. This will involve discussions between test developers, patients, clinicians, hospital administrators, and health ministries to ensure clinical uptake and regular feedback to improve test performance and will ultimately have to include testing in low resource settings as well.

We have stressed the importance of global cooperation in tackling this challenge, using the template of global cooperation on climate change as a starting point. However, we acknowledge that these are challenging times for global treaties. In an era where the rise of nationalist politics is severely undermining international cooperation, it remains to be seen whether, in the long term, the response to the COVID-19 crisis leads to a hardening of international barriers or an increase in cooperation for mutual benefit. However, what is clear is that problems like AMR and pandemics require a globally coordinated response, with nation states accountable to each other as well as to their own citizens for ensuring the health of humanity as a whole. The appetite for global collaboration to help tackle COVID-19 may have opened an opportunity to explore the establishment of early stage “open source” not-for-profit discovery activities. These could potentially feed into development programs, with value built in the data package obtained for submission to regulatory authorities, rather than traditional IP based programs; such approaches have recently been espoused by M4K pharma (https://m4kpharma.com/) and Matthew Todd,^100^ for example.

We were forewarned about the dangers of pandemics from SARS-like coronaviruses,^101^ but with viruses the exact nature and timing of outbreaks is difficult to predict. Policies are already being proposed to minimize the likelihood of COVID-like pandemics in the future; estimates suggest that the cost of such measures (gross) will be around $22–31 billion per year.^102^ With drug resistant microbial infections, the dangers and cost-benefits are more predictable. To cite just one example, the MER based market incentive of $1 billion per antibiotic proposed in the DRIVE-AB report^34^ is *miniscule* in comparison to the potential costs of AMR.^4^ With COVID-19, we are also witnessing the rapid development of complementary push and pull incentives to develop, manufacture, and distribute vaccines across the globe with unprecedented speed.^103^ The progress on the COVID-19 vaccine has underscored the advantages of global cooperation, with research scientists, funders, and vaccine manufacturers joining forces across both HICs and LMICs to overcome the twin challenges of R&D and access.^104^

It is therefore striking that, in the case of antibiotics, market failure is still hindering the development of one of the most significant life-saving measures ever developed by medical science. Solving this must be a top priority for governments. This is an insurance policy that we desperately need. Multisectorial initiatives like the recently launched AMR Action Fund (https://amractionfund.com/) must be supported, expanded, and sustained to maintain a viable antimicrobial development ecosystem. In another promising development, major charities such as the Wellcome Trust are increasingly recognizing the challenges of AMR^105^ and are restructuring their funding priorities to focus further on infectious diseases.

In this Perspective, we have focused primarily on how governments and public organizations can incentivize and support antimicrobial R&D, while ensuring the best outcomes for the public across the globe. The incentives proposed must be sufficient in scale and scope to encourage pharmaceutical companies and their private investors back into the field, while importantly still meeting the access requirements and “One Health” considerations that we propose must be tied to any public investments in the field. A frank and open dialogue between public funders, private investors, health policy makers, patient groups, and pharmaceutical industry leaders will be required to ensure that any incentives (monetary or regulatory) enacted make antimicrobials a viable investment for private companies while meeting public health goals globally. Perhaps a new global awareness of infectious diseases will enable a more effective engagement with civil society and these various public and private institutions, the emergence of a healthy charity sector, and the voices required to help drive change. For many whose lives rely on antimicrobial drugs, the time to act is now.

## Notes

This study did not generate any new data. The data on COVID-19 deaths used in the publication are publicly available at https://www.worldometers.info/coronavirus/ (access date 15th December 2020).

## Abbreviations

COVID: coronavirus disease
SARS: severe acute respiratory syndrome
GDP: gross domestic product
LMICs: low and middle income countries
HICs: high income countries
AMR: antimicrobial resistance
SSIs: surgical site infections
WHO: World Health Organization
R&D: research and development
CAR-T-cell: chimeric antigen receptor T-cell
NPV: net present value
NDM-1: New Delhi metallo-β-lactamase-1
SMEs: small and medium-sized enterprises
MERs: market entry rewards
NGOs: nongovernmental organizations
OMA: options market for antibiotics
CARB-X: Combatting Antibiotic-Resistant Bacteria Biopharmaceutical Accelerator
GARP: Global Antibiotic Resistance Partnership
DRG: diagnosis-related group
HIV: human immunodeficiency virus
SDGs: sustainable development goals
CeBIL: Centre for Advanced Studies in Biomedical Innovation Law
